# Mid-Regional Pro-Adrenomedullin and N-Terminal Pro-B-Type Natriuretic Peptide Measurement: A Multimarker Approach to Diagnosis and Prognosis in Acute Heart Failure

**DOI:** 10.3390/jpm13071155

**Published:** 2023-07-18

**Authors:** Silvia Spoto, Josepmaria Argemi, Roberta Di Costanzo, Juan Josè Gavira Gomez, Nahikari Salterain Gonzales, Stefania Basili, Roberto Cangemi, Antonio Abbate, Luciana Locorriere, Francesco Masini, Giulia Testorio, Rodolfo Calarco, Giulia Battifoglia, Fabio Mangiacapra, Marta Fogolari, Sebastiano Costantino, Silvia Angeletti

**Affiliations:** 1 Diagnostic and Therapeutic Medicine Department, Fondazione Policlinico Universitario Campus Bio-Medico, 00128 Rome, Italy; robertadicostanzo97@gmail.com (R.D.C.); l.locorriere@policlinicocampus.it (L.L.); f.masini@policlinicocampus.it (F.M.); g.testorio@policlinicocampus.it (G.T.); r.calarco@policlinicocampus.it (R.C.); g.battifogliaa@gmail.com (G.B.); s.costantino@policlinicocampus.it (S.C.); 2 Departamento de Medicina Interna, Clinica Universidad de Navarra, Pamplona, 31008 Navarra, Spain; jargemi@unav.es; 3 Departamento de Cardiologìa, Clinica Universidad de Navarra, Pamplona, 31008 Navarra, Spain; jjgavira@unav.es (J.J.G.G.); nsalterain@unav.es (N.S.G.); 4 Department of Translational and Precision Medicine, Sapienza University of Rome, 00161 Rome, Italy; stefania.basili@uniroma1.it (S.B.); roberto.cangemi@uniroma1.it (R.C.); 5 Division of Cardiology, Department of Internal Medicine, Pauley Heart Center, Virginia Commonwealth University, Richmond, VA 23219, USA; aabbatemd@gmail.com; 6 Unit of Cardiovascular Science, University Campus Bio-Medico, 00185 Rome, Italy; f.mangiacapra@policlinicocampus.it; 7 Unit of Laboratory, Fondazione Policlinico Universitario Campus Bio-Medico, 00128 Rome, Italy; m.fogolari@policlinicocampus.it (M.F.); s.angeletti@policlinicocampus.it (S.A.); 8 Research Unit of Clinical Laboratory Science, Department of Medicine and Surgery, University Campus Bio-Medico di Roma, 00184 Rome, Italy

**Keywords:** acute heart failure (AHF), mid-regional pro-adrenomedullin (MR-proADM), N-terminal pro-B-type natriuretic peptide (NT-proBNP), C-reactive protein (CRP), creatinine

## Abstract

Background: Acute heart failure (AHF) is a major cause of hospitalization and mortality worldwide. Early and accurate diagnosis, as well as effective risk stratification, are essential for optimizing clinical management and improving patient outcomes. In this context, biomarkers have gained increasing interest in recent years as they can provide important diagnostic and prognostic information in patients with AHF. Aim and Methods: The primary objective of the present study was to compare the levels of N-terminal pro-B-type natriuretic peptide (NT-proBNP), mid-regional pro-adrenomedullin (MR-proADM), and C-reactive protein (CRP) between patients diagnosed with acute heart failure (AHF) and those without AHF and sepsis. Furthermore, the study aimed to assess the diagnostic and prognostic value of the use of a multimarker approach in AHF patients. To achieve these objectives, a total of 145 patients with AHF and 127 patients without AHF and sepsis, serving as the control group, were consecutively enrolled in the study. Results: Levels of MR-proADM (median: 2.07; (25th–75th percentiles: 1.40–3.02) vs. 1.11 (0.83–1.71) nmol/L, *p* < 0.0001), and NT-proBNP (5319 (1691–11,874) vs. 271 (89–931.5) pg/mL, *p* < 0.0001) were significantly higher in patients with AHF compared to controls, whereas CRP levels did not show significant differences. The mortality rate in the AHF group during in-hospital stay was 12%, and the rate of new re-admission for AHF within 30 days after discharge was 10%. During in-hospital follow-up, Cox regression analyses showed that levels of NT-proBNP > 10,132 pg/mL (hazard ratio (HR) 2.97; 95% confidence interval (CI): 1.13–7.82; *p* = 0.0284) and levels of MR-proADM > 2.8 nmol/L (HR: 8.57; CI: 2.42–30.28; *p* = 0.0009) predicted mortality. The combined use of MR-proADM and NT-proBNP provided significant additive predictive value for mortality and new re-admission for AHF at 30 days after discharge. A logistic regression analysis showed that the presence of NT-proBNP pg/mL > 12,973 pg mL and/or MR-proADM > 4.2 nmol/L predicted hospital re-admission within 30 days (OR: 3.23; CI: 1.05–9.91; *p* = 0.041). Conclusion: The combined assay of MR-proADM and NT-proBNP could be helpful in accurately identifying AHF and in defining prognosis and re-admission for AHF. The complementary use of these biomarkers can provide a useful clinical evaluation of AHF while also orienting clinicians to the pathophysiology underlying heart damage and assisting them in tailoring therapy.

## 1. Introduction

Acute heart failure (AHF) is a serious and potentially life-threatening condition that requires prompt medical attention. It is characterized by the sudden onset or worsening of symptoms and signs of heart failure [1,2,3,4,5,6,7,8]. The prevalence of AHF is relatively high in the adult population, estimated to be around 1–2%. However, the prevalence of AHF increases with age, and it is particularly high in subjects over 70 years of age, reaching up to 10% [9,10,11,12]. According to the literature, in developed countries, the median age of patients with AHF at the time of presentation is around 75 years old [13]. AHF is the leading cause of urgent hospitalization in patients older than 65 years, accounting for 5–10% of all hospitalizations. Unfortunately, AHF is associated with high morbidity and mortality rates. In-hospital mortality rates are around 4%, while mortality rates at 60–90 days after discharge can reach 10%. Furthermore, up to 25–30% of patients with AHF may die within one year after the onset of symptoms [4,13,14,15].

The literature suggests that the 90-day re-admission rate for patients with AHF can reach 25–30%. This high re-admission rate is likely due to the complexity and chronic nature of heart failure, which often requires ongoing management and close monitoring. Additionally, the median length of stay (LOS) for patients with AHF is reported to be around 5.6 days, which highlights the need for efficient and effective management strategies to improve patient outcomes and reduce healthcare utilization [13,15].

Numerous prognostic factors for death and/or hospitalization have been identified in patients with AHF, including clinical variables, long-lasting HF, myocardial remodeling, biomarkers, and cardiovascular and non-cardiovascular comorbidities. However, their clinical applications are limited, and exact risk stratification remains challenging [16,17,18,19,20].

Among biomarkers, the N-terminal pro-B-type natriuretic peptide (NT-proBNP) and C-reactive protein (CRP) have been extensively studied for their correlations with AHF [21,22,23,24,25,26,27,28].

The serum level of NT-proBNP has been recommended by the American Heart Association (AHA) as a diagnostic tool for AHF [1,2,29,30,31]. Additionally, its level has been found to be reliable for the classification of severity, prognosis, and mortality in patients with AHF [1,2,29,30,31].

N-terminal pro-B-type natriuretic peptide is a biologically inert 76-amino acid peptide that is derived from the cleavage of pro-brain natriuretic peptide (NT-proBNP). It is secreted by myocardial cells in response to hemodynamic stress, volume expansion, and pressure load. It promotes diuresis and vasodilation as compensatory mechanisms. The level of NT-proBNP in the blood correlates with the severity of ventricular dysfunction from both clinical and hemodynamic perspectives. In patients with left ventricular dysfunction, levels of NT-proBNP increase more than those of BNP. Additionally, NT-proBNP has a longer plasma half-life (70 min versus 20 min), making it a preferable biomarker to measure [28].

Serum proteins like albumin, C-reactive protein (CRP), interleukin 6, and other inflammatory biomarkers play significant roles in the aging process and the heightened risk of mortality [32,33]. C-reactive protein is among the most extensively studied inflammatory biomarkers. However, data concerning its predictive value in patients with AHF remain limited [34]. A recent biomarker that has been evaluated for its possible correlation with AHF is mid-regional pro-adrenomedullin (MR-proADM); MR-proADM is a 48-amino acid fragment released from the medial region of proADM in a 1:1 ratio with adrenomedullin (ADM), which proportionally reflects ADM levels and activity but has a longer half-life. While the half-life of ADM is 22 min, the stability of MR-proADM is at least 75 days in the absence of clinical changes. Therefore, it can only be measured once at the time of patient hospitalization [35].

Furthermore, MR-proADM was found to be more stable and easier to measure in peripheral blood [35]. The role of ADM biomarkers appears to be compensatory in AHF by counterbalancing the increased peripheral resistance and reducing cardiac preload. Adrenomedullin is a multipotent regulatory peptide with a number of biological activities, acting as a vasodilator, positive inotropic, diuretic, natriuretic, and bronchodilator, and is widely expressed throughout the body, including in the bone, adrenal cortex, kidney, lung, blood vessels, and heart. It is upregulated by hypoxia, inflammatory cytokines, bacterial products, and shear stress [36]. Chronically, ADM also has antihypertrophic, anti-apoptotic, antifibrotic, antioxidant, and angiogenic effects. Moreover, it can serve as a biomarker for illness; in fact, it has been shown that MR-proADM values vary according to the severity and etiology of AHF in patients [36,37,38].

Myocardial injury can cause organ damage through one of three mechanisms: the stretching of myocytes, myocardial damage, or oxidative stress. In congestive heart failure, there is a failure of the ADM system, leading to the inhibition of vasodilation, a significant increase in perfusion, and the inhibition of the renin–angiotensin–aldosterone system (RAAS), which causes vasoconstriction. Additionally, the ADM system protects endothelial integrity, reducing vascular permeability. Without this protection, there is progressive vascular loss, an increased inflammatory state, the activation of the coagulation cascade, and potential systemic organ damage [36,37].

The present study aims to stratify patients with AHF based on levels of NT-proBNP, MR-proADM, CRP, and creatinine at admission, as well as the level of NT-proBNP measured at discharge. The biomarkers will be correlated with clinical severity, 30-day mortality, re-admission for AHF at 30 days, and length of hospital stay, with the goal of guiding the best management of patients and reducing mortality. Additionally, as a secondary outcome, biomarkers will be evaluated in relation to NYHA class [16] and left ventricular ejection fraction for patient stratification.

## 2. Materials and Methods

### 2.1. Study Design

This single-center prospective longitudinal study was conducted between October 2016 and May 2022 at the Diagnostic and Therapeutic Medicine Department of the Fondazione Policlinico Universitario Campus Bio-Medico in Rome, Italy. It was approved by the Local Ethics Committee of the Fondazione Policlinico Universitario Campus Bio-Medico (N° 23.17 TS). All participants provided written informed consent before enrollment, and the study was conducted in accordance with the principles of the Declaration of Helsinki. 

The study included patients aged 18 years or older with AHF classified as NYHA I–IV. In the same period, patients who were admitted to the hospital without AHF and sepsis and presented similar comorbidities (such as arterial hypertension, chronic obstructive pulmonary disease, chronic kidney disease, diabetes mellitus, a smoking habit, dyslipidemia, and atrial fibrillation) were enrolled as control group.

Patients with end-stage renal disease (ESRD), a history of angioedema, pregnancy, lactation, or active inflammatory or infectious diseases were excluded. All subjects included in the study were consecutively enrolled.

The diagnosis of AHF was made based on the clinical guidelines provided by the European Society of Cardiology in 2016 and 2021 [1,2].

Upon admission, patients underwent a medical history interview and a complete physical examination. Vital signs were monitored, and both an electrocardiogram and a transthoracic echocardiogram were performed.

The biomarkers, NT-proBNP, MR-proADM, CRP, and creatinine, were measured at the time of admission. 

Furthermore, in the AHF patients, a follow-up measurement of NT-proBNP was obtained at the time of discharge. Information about the length of hospitalization, re-admission due to heart failure, and 30-day mortality following discharge were collected and documented.

### 2.2. Biomarker Measurement

Plasma concentrations of NT-proBNP, CRP and creatinine were measured via the chemiluminescence method using Alinity C (Autoanalyzer Abbott). The blood concentrations of MR-proADM were measured with an automatic Kryptor analyzer, using a time-resolved amplified emission method (Kryptor; Brahms AG; Hennigsdorf, Germany) with commercially available immunoassays.

### 2.3. Statistical Analysis

Categorical variables are reported as counts and percentages, and continuous variables are reported as means ± SDs or medians and [25th–75th percentiles]. Differences between percentages were assessed via chi-square or Fisher’s exact tests. Student’s unpaired *t*-tests and an analysis of variance were used for normally distributed continuous variables. Appropriate nonparametric tests were used for all other variables. 

The diagnostic sensitivity and specificity of the biomarkers were evaluated by constructing receiver operating characteristic (ROC) curves and determining the optimal cut-off values. The correlations between biomarker levels and NYHA class were analyzed using Spearman’s rank correlation.

The bivariate and multivariate effects of prognostic factors on the occurrence of hospital re-admission in AHF patients were also assessed by means of logistic regression models. Wald confidence intervals and tests for odds ratios (ORs) and adjusted odds ratios were computed based on the estimated standard errors. The stochastic level of entry into the multivariable model was set at 0.10.

To estimate the cumulative mortality incidence after grouping the population, the Kaplan–Meier product–limit estimator was utilized. The log-rank test was employed to formally compare survival curves. To determine the adjusted relative risks of outcome events for each clinical variable, a Cox proportional hazards analysis was conducted.

A *p*-value less than 0.05 was considered statistically significant. All tests were two-tailed. Data were analyzed using the following computer statistical software: SPSS, IBM Corp, released 2020; IBM SPSS Statistics for Windows, Version 27.0, Armonk, NY, USA, IBM Corp; and MedCalc: MedCalc Statistical Software, version 12.7.0.0 (MedCalc Software Ltd. Ostend, Belgium; https://medcalc.org; accessed on 2 July 2023).

## 3. Results

The patient population with AHF consisted of 145 subjects (52% men), with a median age of 82 years [75–87], while the control population (non-AHF, non-septic patients) was composed of 127 subjects (55% men), with a median age of 76 years [82–88] (Table 1).

The pathogenesis of heart failure in the AHF group was hypertensive (89%), ischemic (28%), degenerative (57%), and dilated (19%). In patients with AHF, the median NYHA class was III. The median LVEF was 50 (40–57)%. 

Table 1 presents the demographic and laboratory characteristics of the population under study. The two groups were comparable for sex and all the considered comorbidities. Nevertheless, age was significantly higher in the AHF patients.

Patients with AHF had significantly higher median values of NT-proBNP when compared to the control group (*p* < 0.0001). Similarly, the study observed comparable trends for MR-proADM, CRP, and serum creatinine (*p* < 0.0001). 

**Figure 1:**
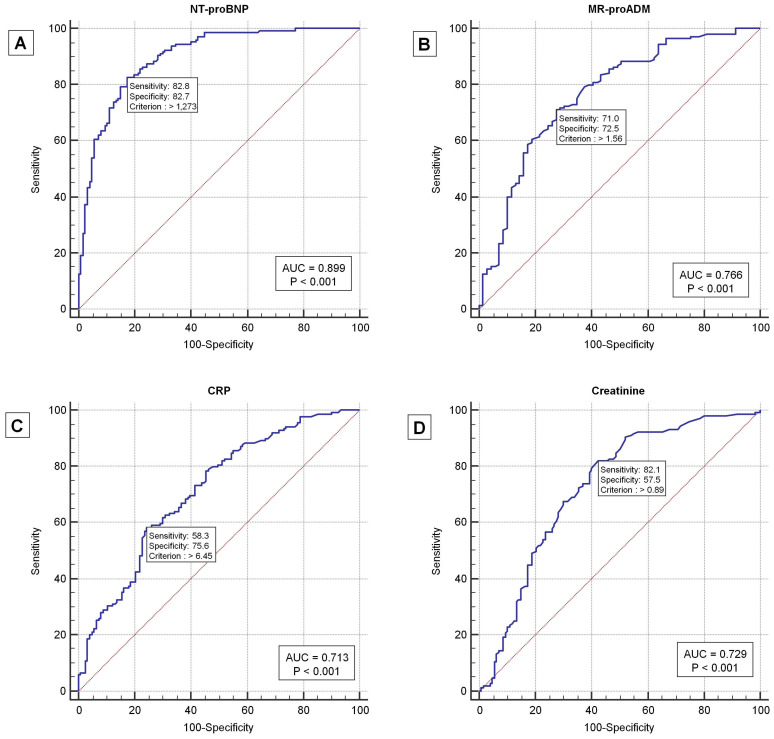
ROC curves for NT-proBNP (**A**), MR-proADM (**B**), CRP (**C**), and creatinine (**D**) in AHF patients compared to the control group.

As expected, in patients with AHF, NT-proBNP values decreased at the time of discharge compared to those measured at admission (3405 [1061–6820] vs. 5319 [1691–11,874] pg/mL, *p* = 0.0025). In the analysis of the ROC curve, the following biomarker cut-offs were found to be useful for diagnostics and prognostics: NT-proBNP > 1273 pg/mL, MR-proADM > 1.56 nmol/L, CRP > 6.45 mg/dL, and creatinine > 0.89 mg/dL (Table 2, Figure 1).

In patients with AHF, the median length of stay (LOS) was significantly longer than in the control group (*p* = 0.02) (Table 1).

The mortality rate in the AHF group was 12% (17 out of 145), and the 30-day re-hospitalization rate for AHF patients was 10%.

**Figure 2:**
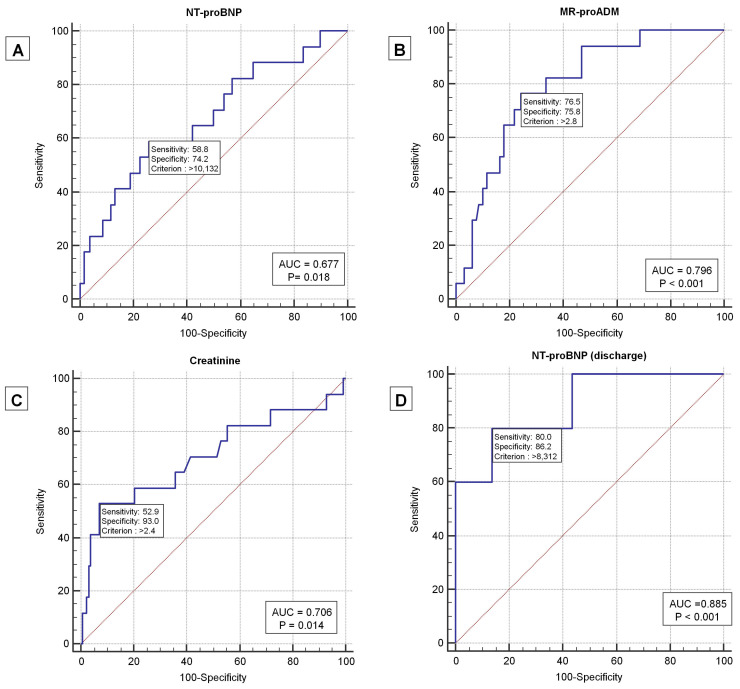
ROC curves for NT-proBNP (**A**), MR-proADM (**B**), creatinine (**C**), and NT-proBNP at discharge (**D**) in relation to mortality in AHF patients.

The NT-proBNP cut-offs of >10,132 pg/mL at admission and >8312 pg/mL at discharge were identified as correlated with mortality. Additionally, levels of MR-proADM > 2.8 nmol/L and creatinine > 2.4 mg/dL showed significant correlations with mortality, as depicted in Table 3 and Figure 2.

When the baseline NT-proBNP level exceeds 10,132 pg/mL and the level of MR-proADM is above 2.8 nmol/L, combining the two markers results in a PPV for mortality of 48.5%, which is higher than the individual biomarkers’ PPVs (23.3% for NT-proBNP and 29.53% for MR-proADM) (Table 4). Additionally, if a patient has an NT-proBNP value > 8312 pg/mL at discharge, in addition to the same values of NT-proBNP and MR-proADM at baseline, the PPV for mortality increases to 85% (Table 4).

**Figure 3:**
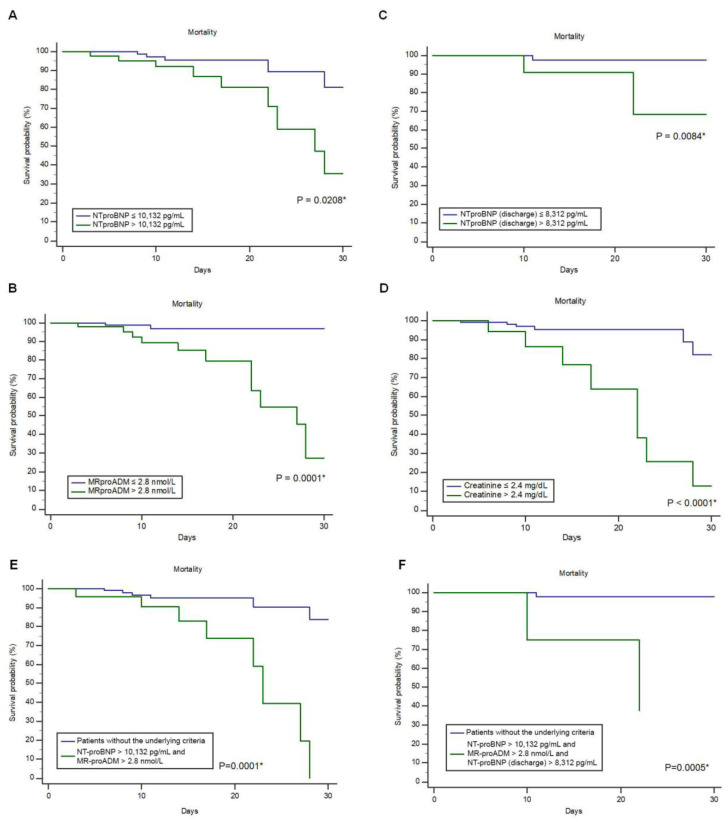
KM curves for 30-day mortality of NT-proBNP (**A**), MR-proADM (**B**), and NT-proBNP patients at discharge (**C**), creatinine (**D**), combination of NT-proBNP and MR-proADM (**E**), and combination of NT-proBNP, MR-proADM, and NT-proBNP at discharge (**F**) * Log-rank test.

A univariate Cox regression analysis showed that levels of NT-proBNP > 10,132 pg/mL (hazard ratio (HR) 2.97; 95% confidence interval (CI): 1.13–7.82; *p* = 0.0284), MR-proADM > 2.8 nmol/L (HR: 8.57; CI: 2.42–30.28; *p* = 0.0009), and NT-proBNP > 8.312 pg/mL at discharge (HR: 11.25; CI: 1.24–102.50; *p* = 0.0326) and serum creatinine > 2.4 mg/dL (HR: 8.60; CI: 3.06–24.17; *p* < 0.0001) predicted mortality in AHF patients. Moreover, the combination of NT-proBNP > 10,132 and MR-proADM > 2.8 nmol/L predicted mortality with an HR of 2.61 (CI: 1.52–4.49; *p* = 0.0005), and the combination oof NT-proBNP > 10,132, MR-proADM > 2.8 nmol/L, and NT-proBNP > 8.312 pg/mL at discharge predicted mortality with an HR of 2.80 (CI: 1.24–6.28; *p* = 0.0132) (Figure 3).

### 3.1. Re-Hospitalization within 30 Days

An NT-proBNP cut-off level of >12,973 pg/mL at baseline demonstrates a correlation with new re-admission for AHF within 30 days after discharge, with a positive predictive value (PPV) of 15.6%. However, if an MR-proADM value > 4.2 nmol/L is detected at baseline (with a PPV of 23.5%), the combined PPV increases to 35%.

A logistic regression analysis showed that the presence of NT-proBNP pg/mL > 12,973 pg ml and/or MR-proADM > 4.2 nmol/L predicted hospital re-admission at 30 days (OR: 3.23; 95% CI: 1.05–9.91; *p* = 0.041).

### 3.2. Re-Hospitalizations after 30 Days

Twenty-seven AHF patients were re-admitted to the hospital after 30 days.

A logistic analysis that incorporated the aforementioned biomarkers (NT-proBNP at admission and at discharge and MR-proADM) and comorbidities showed a significant increased risk of re-hospitalization only for diabetes, which was associated with a four times higher risk of re-hospitalization compared to non-diabetic AHF patients (odds ratio (OR) = 4.21; 95% CI 1.38–12.87). 

### 3.3. Biomarkers in Combination with NYHA Class Stratification in Patients with AHF

Among patients with AHF in NYHA class I, the median baseline NT-proBNP value was 6185 [0.08–12,401] pg/mL, while the median NT-proBNP value at discharge was 5448.5 [3327–6038] pg/mL. Additionally, the median baseline MR-proADM value was 1.99 [0.98–2.3] nmol/L, the CRP value was 6.38 [2.2–11.2] mg/dL, and the creatinine value was 1.32 [1.02–2.02] mg/dL.

In patients with AHF in NYHA class II, the median baseline NT-proBNP value was 3896 [0.00–10,453] pg/mL, while the median NT-proBNP value at discharge was 1486 [673–6768] pg/mL. The median baseline MR-proADM value was 1.90 [1.2–3] nmol/L, the CRP value at baseline was 11.2 [2–53] mg/dL, and the creatinine value at baseline was 1.21 [0.92–1.55] mg/dL.

For patients with AHF class NYHA III, the median baseline value of NT-proBNP was 5483 [2136–12,255] pg/mL, which decreased to 3405 [1153–7823] pg/mL at discharge. The median baseline value of MR-proADM was 2.07 [0.59–3] nmol/L, while those of CRP and creatinine were 8.8 [2–26] mg/dL and 1.14 [0.93–1.70] mg/dL, respectively.

There was no significant correlation observed between NT-proBNP values, MR-proADM, CRP, and creatinine with NYHA classes I–IV.

### 3.4. Left Ventricular Ejection Fraction (LVEF) Patient Stratification and Biomarkers

**Figure 4:**
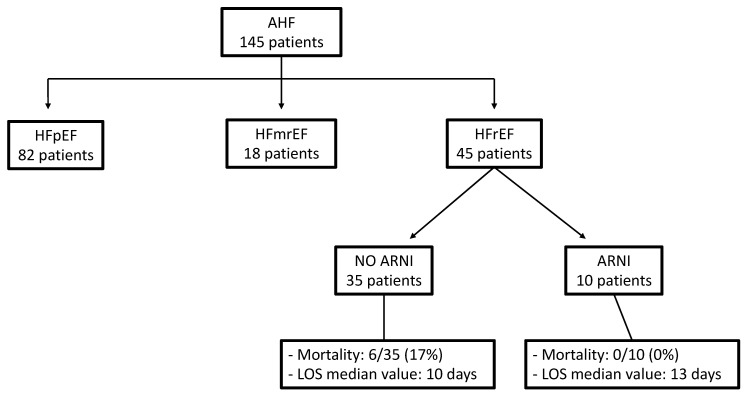
Stratification of AHF patients in agreement with LVEF. AHF—acute heart failure; ARNI—angiotensin receptor–neprilysin inhibitor; HFmrEF—heart failure with mildly reduced ejection fraction; HFpEF—heart failure with preserved ejection fraction; HFrEF—heart failure with reduced ejection fraction; LOS—length of stay; LVEF—left ventricular ejection fraction.

The study population was stratified based on their LVEF (Figure 4). Out of 145 patients with AHF, 10 subjects (6.89%) with reduced EF (HFrEF) were treated with sacubitril/valsartan (ARNI) therapy. Among this group of patients, the median LVEF was significantly lower than in the group that did not receive ARNI therapy (*p* = 0.0001). However, there was no substantial difference between the two groups in terms of the median values of all biomarkers measured at baseline and NT-proBNP values at discharge (Table 5). 

Interestingly, a significant difference in the mortality rate was observed, with a 0% mortality rate in the ARNI group compared to 12% in the non-ARNI group of patients with AHF (Figure 4).

The mortality rate in the AHF population was 17 out of 145 patients (12%). Out of the 45 patients in the HFrEF group, 6 patients (13%) died, and all these deaths were among the ARNI-untreated HFrEF group (Figure 4).

The length of stay (LOS) for the ARNI HFrEF group was significantly longer compared to the non-ARNI HFrEF group (*p* = 0.0265), as shown in Figure 4.

**Figure 5:**
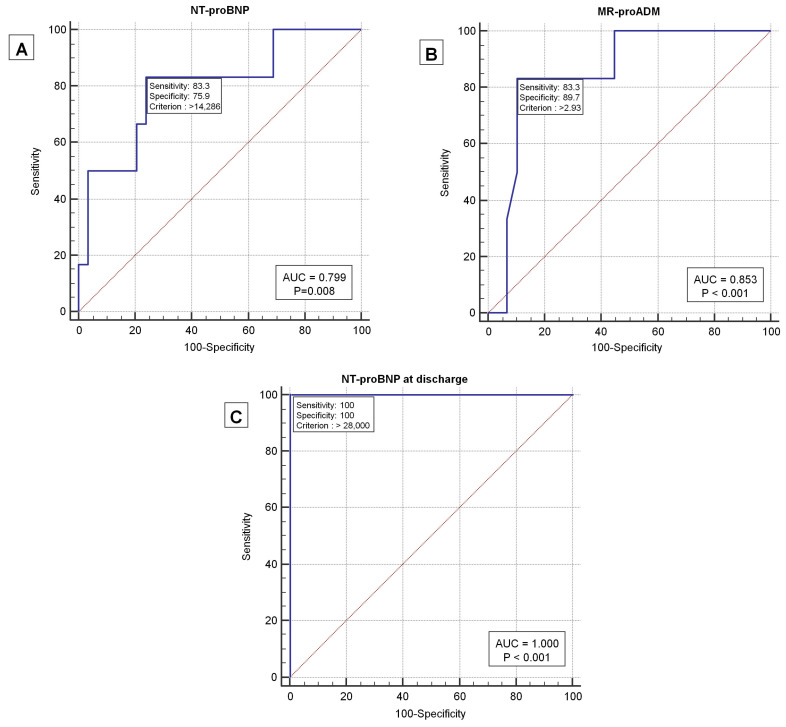
ROC curve of NT-proBNP at time 0 and mortality in ARNI patients (**A**); ROC curve of MR-proADM at time 0 and mortality in ARNI patients (**B**); ROC curve of NT-proBNP at discharge and mortality in non-ARNI HFrEF patients (**C**).

At baseline, among the 35 non-ARNI HFrEF patients, the ROC curve highlighted cut-off values for mortality of NT-proBNP > 14,286 pg/mL, with a PPV of 41.6% and an NPV of 95.6%, and MR-proADM > 2.93 nmol/L, with a PPV of 62.5% and an NPV of 96.3% (Figure 5). The combination of these two biomarkers in series increased the PPV up to 85%. Moreover, if an NT-proBNP value > 28,000 pg/mL at discharge is also found, the PPV for mortality increases from 85% to 99% (Figure 5).

## 4. Discussion

Acute heart failure is a clinical syndrome that affects approximately 5.8 million people out of an estimated 23 million worldwide. This number is expected to increase due to the increasing age of the population and the improved treatments for cardiovascular diseases that have reduced mortality but can lead to the development of AHF [3].

In addition to classical diagnostic methods for AHF, biomarker measurement has been introduced as it provides a more objective and quicker evaluation strategy. Among the biomarkers used, troponin measurement is recommended during myocardial infarction as an index of necrosis in an acute setting. However, its role in risk stratification in individuals with HF in a non-acute setting is still debated [39,40].

Other biomarkers, which have been proposed through a multi-panel approach, appear to be promising for the diagnosis, progression, and management of AHF [41]. This may enable a personalized therapy approach for each patient, promoting the concept of precision medicine.

The clinical use of MR-proADM in comparison to NT-proBNP is supported by the fact that NT-proBNP is primarily a hemodynamic marker that indicates the degree of myocyte strain response caused by hemodynamic stress and is affected by short-term functional overload. On the other hand, MR-proADM is a marker of disease that reflects the level of oxidative stress and thus the severity of the disease.

In our study, we found that NT-proBNP levels greater than 1273 pg/mL, MR-proADM levels exceeding 1.56 nmol/L, CRP levels above 6.45 mg/dL, and creatinine levels higher than 0.89 mg/dL were able to identify patients with AHF.

Except for CRP, all the biomarkers evaluated in this study were found to be correlated with mortality. Specifically, NT-proBNP values > 10,132 pg/mL, MR-proADM values > 2.8 nmol/L, and creatinine values > 2.4 mg/dL at admission were able to identify patients with AHF who were at the highest risk of mortality.

There was no demonstrated correlation between the levels of NT-proBNP, MR-proADM, CRP, and creatinine and NYHA class. Additionally, no correlation was found between LVEF and either mortality or 30-day re-admission for AHF.

It is noteworthy that there is a high negative predictive value (NPV) associated with the measurement of NT-proBNP, MR-proADM, and creatinine levels at admission. If any of these markers are lower than the established cut-off, it is unlikely that the patient will experience adverse outcomes. MR-proADM was found to have the highest NPV at admission among these markers. In addition, the NT-proBNP level measured at discharge was found to be correlated with mortality, with a value below 8312 pg/mL excluding adverse outcomes at 30 days in almost 99% of cases.

None of the biomarkers were found to be correlated with the need for re-hospitalization for AHF beyond 30 days. The NT-proBNP values at hospitalization were higher compared to the values at discharge, indicating treatment efficacy. However, persistently high NT-proBNP values at discharge were found to be associated with a worse prognosis, in agreement with the existing literature [27,31].

It is worth noting that a significant correlation was observed between patients with diabetes and re-hospitalization beyond 30 days. In the study group, we found a longer median length of stay compared to the literature (11 days versus 5.6 days) but a lower mortality rate (12% vs. 16.5–34% reported in the literature) and a 30-day re-hospitalization rate for AHF of 10% (vs. 25–30% reported in the literature at 90 days) [39,40,41,42,43].

It is worth noting that the mortality rate in the HFrEF population was 6 out of 45 patients (13%), all of whom belonged to the group that could not receive ARNI, while no patients in the ARNI group died. Moreover, the positive effect of this drug was confirmed by a net increase in the mean LVEF of approximately 3% during follow-up for these patients. As for the longer duration of hospital stay observed in the ARNI group compared to the non-ARNI HFrEF group, this could be attributed to various factors, such as the need for washout from standard therapy, a phase of greater hemodynamic compensation for the inclusion of ARNI, and the time required for drug tolerance assessment.

It should be highlighted that for patients treated with ARNI, AHF triggers the release of various peptides, such as BNP, ADM, bradykinin, and angiotensin II, through the natriuretic peptide system and the activation of the RAAS [44,45]. These peptides are broken down by neprilysin, which decreases vasoconstriction, sodium retention, and maladaptive remodeling. Therefore, while BNP and ADM, along with their more stable and easily detectable counterpart MR-proADM, reflect the effect of ARNI on the heart, NT-proBNP is not broken down by neprilysin and reflects the hemodynamic state [46,47].

In the AHF population (HFpEF, HFmrEF, and HFrEF), NT-proBNP shows a statistically significant reduction at discharge. However, this reduction is not statistically significant in the HFrEF population, nor in the population that received ARNI or the population that could not receive it. Since NT-proBNP is not a substrate of neprilysin, its reduction only indicates the hemodynamic response to treatment. On the other hand, regardless of LVEF or treatment with ARNI, the dosage of MR-proADM at admission indicated an equal severity of oxidative heart damage in the entire AHF population. The half-life of MR-proADM, which remains stable for up to 75 days in the absence of new acute insults, is the reason why further dosing of this biomarker is not needed at discharge [35,43].

Therefore, in our study, we found that MR-proADM had comparable diagnostic accuracy to the gold-standard NT-proBNP in AHF patients [1,41]. Moreover, the combined use of MR-proADM and NT-proBNP at admission and discharge significantly increased the prognostic value and predictive value for mortality and new re-admission for AHF within 30 days of discharge [48]. However, contrary to the recent literature, CRP did not demonstrate a correlation with mortality [41]. It is worth noting that the combined use of multiple biomarkers provides diverse information that reflects different pathophysiological pathways of severe cardiac injury, which can facilitate personalized and targeted therapy for patients [41,49].

The MR-proADM biomarker indicates cardiovascular stress, whereas NT-proBNP is indicative of hemodynamic disturbances and myocardial strain. CRP, on the other hand, measures the degree of inflammation resulting from myocyte stress.

Additionally, the costs and turnaround times of measuring MR-proADM and NT-proBNP are comparable. 

### 4.1. Strengths of the Study

This study has demonstrated the potential benefits of utilizing multiple biomarkers in conjunction with clinical parameters to identify and predict the prognosis and re-admission risk of patients with AHF. By combining biomarkers such as NT-proBNP, MR-proADM, CRP, and creatinine with traditional clinical parameters, the study suggests that a more comprehensive and accurate assessment of AHF patients can be achieved. The incorporation of multiple biomarkers provides supplementary information that goes beyond what can be obtained from clinical parameters alone. These biomarkers could reflect various physiological processes or pathological conditions associated with AHF, thereby offering valuable insights into the severity, progression, and future outcomes of the disease.

### 4.2. Study Limitations

The primary limitation of this study pertains to the selection of the control group. There was a statistically significant difference in age between the control group and the study group, and it is widely recognized that age is a significant factor in the occurrence of acute heart failure. Furthermore, the study acknowledges the need for more comprehensive data, a larger sample size of enrolled patients, a longer duration of follow-up, and the expansion of the study to multiple centers to validate its potential clinical utility.

## 5. Conclusions

The combined assay of NT-proBNP, MR-proADM, and CRP could be helpful in accurately identifying congestive heart failure. The values of MR-proADM and CRP at diagnosis were found to be significantly higher in patients with AHF than in controls. High levels of NT-proBNP, MR-proADM, and CRP can identify patients with AHF, but only NT-proBNP and MR-proADM can also express prognosis, while elevated CRP levels do not help in this regard. This study suggests a possible diagnostic and prognostic role of MR-proADM in acute heart failure. The validity of incorporating MR-proADM in addition to NT-proBNP stems from their complementary roles in assessing different aspects of the disease. While NT-proBNP reflects the myocardial response to hemodynamic stress and overload from a functional perspective, MR-proADM provides insights into the extent of oxidative stress and the severity of the disease. By considering both biomarkers, a more comprehensive evaluation of the pathological processes underlying acute heart failure can be achieved. Therefore, their complementary use can be useful for the clinical evaluation of heart failure while also providing guidance as to the pathophysiology underlying heart damage and tailoring therapy. However, this study has limitations that should be considered. These limitations include the need for more comprehensive data, a larger sample size, and longer follow-up periods and the inclusion of a multicenter design to validate the potential clinical utility of the findings.

## Figures and Tables

**Table 1:** Demographic characteristics, clinical features, and biomarkers of AHF patients and non-AHF, non-septic patients.

Variables	AHF n = 145	Non AHF Non Septic n = 127	*p* Value
Age, yrs.	80 ± 11	75 ± 12	<0.0001
Male	76 (52)	70 (55)	0.4075
Anamnestic variable			
Arterial hypertension	131 (90)	107 (84)	0.1297
COPD	52 (36)	42 (33)	0.6289
Chronic kidney disease	63 (43)	51 (40)	0.5830
Diabetes mellitus	52 (36)	41 (32)	0.5345
Current smoker or ex-smoker	79 (54)	58 (46)	0.1467
Dyslipidemia	79 (54)	63 (50)	0.4218
Atrial fibrillation	97 (67)	74 (58)	0.1418
BMI, kg/m^2^	26 [23.5–29.0]	26 (23.0–29.0)	0.8463
BMI ≥ 30 kg/m^2^ BMI ≤ 25 kg/m^2^	23 (16) 54 (37)	20 (16) 49 (38)	0.9795 0.8200
NT-proBNP, pg/mL	5319 [1691–11,874]	271 [89–931.5]	<0.0001
MR-proADM, nmol/L	2.07 [1.40–3.02]	1.11 [0.83–1.71]	<0.0001
CRP, mg/dL	8.8 [2.3–29.0]	1.85 [0.45–6.50]	<0.0001
Creatinine, mg/dL	1.26 [0.9–1.71]	0.81 [0.70–1.15]	<0.0001
LOS	11 [8–14.5]	9 [7–13]	0.02

Legend: AHF—acute heart failure; BMI—body mass index; COPD—chronic obstructive pulmonary disease; CRP—C-reactive protein; proADM—mid-regional pro-adrenomedullin; NT-proBNP—N-terminal pro-B-type natriuretic peptide; LOS—length of stay. Data are presented as means + SDs, medians [25th–75th percentiles], or count (%).

**Table 2:** Diagnostic accuracy of biomarkers in AHF patients.

Biomarkers	Cut-Off Value	AUC%	*p* Value	PPV%	NPV%	Sens%	Spec%	LR+	LR−	Prev%
MR-proADM, nmol/L	1.56	76.6	<0.0001	84	54	71	72	2.58	0.40	67.76
NT-proBNP, pg/mL	1273	89.9	<0.0001	84.9	81	82.76	82.68	4.78	0.21	53.31
CRP, mg/dL	6.45	69	<0.0001	72	60	56.25	75.59	2.30	6.58	53.14
Creatinine, mg/dL	0.89	0.73	<0.0001	68.8	73.7	82	57.5	1.93	0.31	53.31

Legend: MR-proADM—mid-regional pro-adrenomedullin; NT-proBNP—N-terminal pro-B-type natriuretic peptide; CRP—C-reactive protein; AUC—area under the curve; PPV—positive predictive value; NPV—negative predictive value; Sens—sensitivity; Spec—specificity; LR+—positive likelihood ratio; LR−—negative likelihood ratio; Prev%—prevalence.

**Table 3:** Prognostic accuracy of biomarkers in AHF patients.

Biomarkers	Cut-Off Value	AUC%	*p* Value	PPV%	NPV%	Sens%	Spec%	LR+	LR−	Prev%
MR-proADM, nmol/L	2.8	0.80	<0.0001	29.53	96	76.47	75.78	3.16	0.31	11.72
NT-proBNP, pg/mL	10,132	0.68	0.018	23.3	93	58.8	74.2	2.28	0.55	11.72
NT-proBNP, pg/mL at discharge	8312	88.5	<0.001	26.3	98.6	80	86	5.82	0.23	5.88
Creatinine, mg/dL	2.4	0.71	0.0138	50	94	53	93	7.53	0.51	11.72

Legend: MR-proADM—mid-regional pro-adrenomedullin; NT-proBNP—N-terminal pro-B-type natriuretic peptide; CRP—C-reactive protein; AUC—area under the curve; PPV—positive predictive value; NPV—negative predictive value; Sens—sensitivity; Spec—specificity; LR+—positive likelihood ratio; LR−—negative likelihood ratio; Prev%, prevalence.

**Table 4:** Positive predictive value (PPV) of combined biomarker assay (NT-proBNP and MR-proADM) as predictor of mortality.

Biomarkers	Cut-Off	PPV%	Combined PPV %	
NT-proBNP, pg/mL	>10,132	23.3	48.5	85
MR-proADM, nmol/L	>2.8	29.53		
NT-proBNP, pg/mL at the discharge	>8312	26.3		

**Table 5:** Biomarker median values in HFrEF patients (ARNI and non-ARNI).

Biomarkers	HFrEF ARNI (N = 10)	HFrEF non-ARNI (N = 35)	*p* Value
NT-proBNP, pg/mL	8649.5 [3393–11,229.0]	7918.0 [2845.0–33,531.0]	0.4206
NT-proBNP, pg/mL at the discharge	2467.5 [1562.5–4622.0]	5672.0 [1897.0–9779.0]	0.1572
MR-proADM, nmol/L	1.89 [1.11–2.80]	2.01 [1.61–2.93]	0.4205

Data are expressed as medians [25th–75th percentiles].

## Data Availability

The data presented in this study are available upon request from the corresponding author.

## Abbreviations

Acute heart failure (AHF); angiotensin receptor-–neprilysin inhibitor (ARNI); areas under the curve (AUC); C-reactive protein (CRP); heart failure with mildly reduced ejection fraction (HFmrEF); heart failure with preserved ejection fraction (HFpEF); heart failure with reduced ejection fraction (HFrEF); length of stay (LOS); left ventricular ejection fraction (LVEF); mid-regional pro-adrenomedullin (MR-proADM); N-terminal pro-B-type natriuretic peptide (NT-proBNP); receiver operating characteristic (ROC).
